# The impact of adjuvant radiotherapy on overall survival in spinal low-grade gliomas: a propensity score-matched analysis

**DOI:** 10.1007/s11060-024-04880-3

**Published:** 2024-11-11

**Authors:** Victor Gabriel El-Hajj, Sruthi Ranganathan, Harry Hoang, Abdul Karim Ghaith, Mohamad Bydon, Adrian Elmi-Terander

**Affiliations:** 1 Department of Clinical Neuroscience, Karolinska Institutet, Stockholm, Sweden; 2 Department of Medicine, University of Cambridge, Cambridge, UK; 3 Faculty of Biology and Medicine, University of Lausanne, Lausanne, Switzerland; 4 Mayo Clinic Neuro-Informatics Laboratory, Mayo Clinic, Rochester, MN USA; 5 Department of Neurological Surgery, Mayo Clinic, Rochester, MN USA; 6 Capio Spine Center Stockholm, Löwenströmska Hospital, Upplands-Väsby, Sweden

**Keywords:** Spinal low-grade glioma, Spinal astrocytoma, Surgery, Adjuvant, Radiotherapy, Radiation

## Abstract

**Introduction:**

Spinal low-grade gliomas (sLGGs) are a group of tumors that arise from glial cells in the spinal cord. Current evidence supporting the use of adjuvant radiotherapy for the management of sLGG is lacking. We hence aimed to compare overall survival rates in patients receiving surgery alone with those receiving surgery with adjuvant radiotherapy.

**Methods:**

The NCDB, a large, nationwide, US-based cancer registry was used. Relevant cases were identified using the following ICD-O-3 histological codes: 9382, 9384, 9400, 9410, 9411, 9420, 9421, 9424, 9425, and 9450, along with the ICD-O-3 topographical codes for spinal meninges (C70.1) and spinal cord (C72.0), excluding spinal ependymomas. Overall survival was the primary outcome. Propensity score matching 1:1 was used to balance the cohorts prior to Kaplan-Meier survival analysis.

**Results:**

A total of 552 patients were included in the study, with 440 in the surgery alone group and 156 in the surgery with adjuvant radiotherapy group. Patients in the surgery with adjuvant radiotherapy group were significantly older (median age 40.0 vs. 24.0 years, *p* < 0.001), and exhibited higher proportions of WHO grade 2 tumors (*p* < 0.001). Adjuvant chemotherapy was more frequently administered in the surgery with adjuvant radiotherapy group (23% vs. 7%, *p* < 0.001). Overall, adjuvant radiotherapy was not associated with improved survival, with a significantly higher mortality in the radiotherapy group before propensity score matching (*p* < 0.0001). After matching, the difference in overall survival was no longer significant (*p* = 0.11).

**Conclusion:**

This study found no significant overall survival benefit associated with the use of adjuvant radiotherapy for spinal low-grade gliomas (sLGG). Although patients who received adjuvant radiotherapy initially demonstrated higher mortality rates, this difference was largely due to confounding factors such as more advanced disease in this group.

**Supplementary Information:**

The online version contains supplementary material available at 10.1007/s11060-024-04880-3.

## Introduction

Low-grade gliomas (LGGs) are a group of primary CNS tumors that arise from glial cells in the central nervous system. Classified as Grade 1 or 2 by the World Health Organization (WHO), these tumors are typically slow-growing and less aggressive compared to their high-grade counterparts. Despite their relatively indolent nature, LGGs infiltrate surrounding neural tissue, complicating their management. In the spinal cord, gliomas are exceedingly rare. While they make up about 25% of all intramedullary spinal tumors [1, 2], they occur at an incidence of 0.22 per 100,000 [3]. While the management of low-grade gliomas of the brain has been extensively studied [4], the same cannot be said about the spinal ones. Consequently, the mainstay of treatment for spinal LGG (sLGG) is often inferred from brain gliomas, and typically involves surgery and adjuvant radiotherapy, with or without chemotherapy. Nonetheless, existing evidence indicates that spinal cord gliomas differ significantly from cranial gliomas in their biological and molecular characteristics. Consequently, inferring treatment strategies for spinal gliomas based on approaches used for brain gliomas may be inappropriate and could overlook distinctions that are essential for effective management [5]. While surgery is well-established as a primary treatment modality [6], evidence supporting the use of adjuvant radiotherapy, particularly for the management of sLGG, remains limited. Using a large nationwide cancer database, the current study aimed to address this gap, by comparing overall survival rates in patients receiving surgery alone with those receiving surgery with adjuvant radiotherapy.

## Methods

### Database and ethics

The National Cancer Database (NCDB) was reviewed to identify patients diagnosed with spinal low-grade gliomas (sLGG) between 2004 and 2017. The NCDB is one of the largest cancer registries in the United States sourced from hospital registry data collected in more than 1,500 Commission on Cancer-accredited facilities and covering close to 75% of cases in the country [7]. Relevant cases were identified using the following ICD-O-3 histological codes: 9382, 9384, 9400, 9410, 9411, 9420, 9421, 9424, 9425, and 9450, along with the ICD-O-3 topographical codes for spinal meninges (C70.1) and spinal cord (C72.0). Ependymomas were excluded from this study as they had been analyzed separately in prior research [8].This study adhered to all ethical standards, and approval from the Mayo Institutional Review Board was waived because the NCDB only contains de-identified data. Since the data comes from a registry, informed consent was not required.

### Variables and primary outcome

Baseline characteristics were collected from the NCDB, including patient-specific details such as age, sex, race, ethnicity, Charlson-Deyo comorbidity index, income quartile, insurance status, type of treating facility, and distance to the facility. Additionally, tumor-related factors such as WHO grade, histological subtype, tumor size (in mm), and metastatic status were documented. Data related to treatment were also gathered, including the type of treatment, extent of tumor resection, time to initiation and duration of radiotherapy, number of fractions, and radiation doses (in cGy). The primary outcome of the study was overall survival.

### Statistics

The Shapiro-Wilk test was used to evaluate the normality of the data. As all continuous data significantly deviated from a normal distribution pattern (Shapiro-Wilk test p value < 0.05), it is presented as a median with interquartile range (IQR) and categorical data as numbers (proportion). The Mann-Whitney U, Chi squared, or Fisher’s exact test were used for between-group comparisons, as appropriate. Propensity score was employed to create balanced comparison groups based on the following covariates: age, Charlson-Deyo comorbidity index, WHO grade, histological subtype, extent of tumor resection, and use of adjuvant chemotherapy. These factors were selected based on clinical relevance, availability in the NCDB, and their potential impact on overall survival outcomes. Despite incomplete data for extent of resection, the variable was still incorporated into the model, in an attempt to adjust for this covariate to the greatest extent possible, ensuring a more comprehensive analysis. For that the `MatchIt` package in R was utilized to perform a 1:1 matching, leveraging the “k-nearest” matching method, with a caliper of 0.2. After matching, standardized mean difference comparison and Love plots were performed to ensure a balanced distribution of the covariates. Kaplan-Meier survival analysis was conducted on both pre- and post-matched cohorts to determine the overall survival over follow-up time. Statistical significance was set at *p* < 0.05 All analyses were conducted using the statistical software program R version 4.0.5.

## Results

### Patient demographics and clinical characteristics

**Fig. 1:**
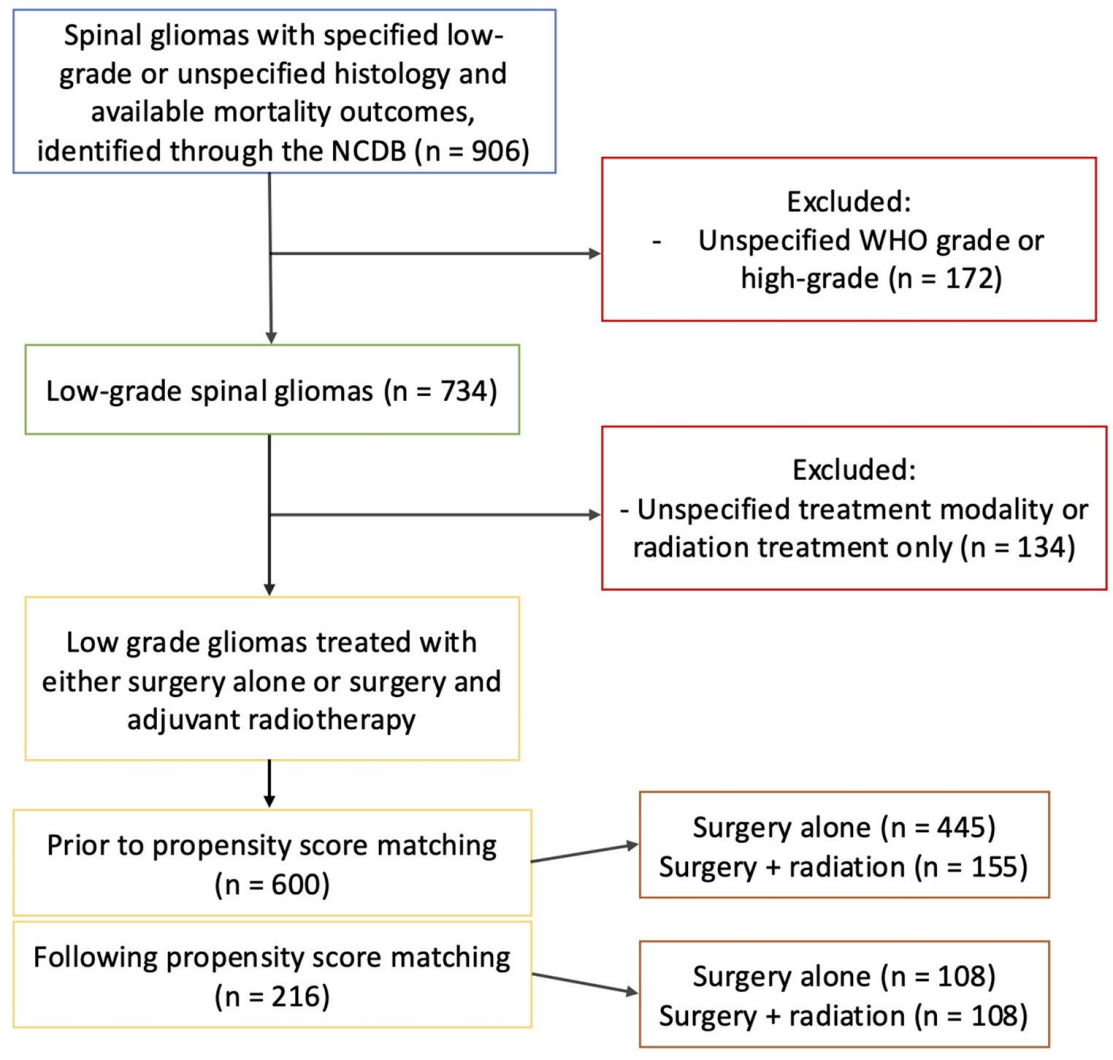
Flowchart illustrating the selection of patients from the NCDB database

A total of 600 patients with sLGG were included in the study (Fig. 1), with 445 undergoing surgical treatment alone and 155 undergoing surgical treatment with adjuvant radiotherapy (Tables 1, 2).

**Table 1:** Baseline demographics and tumor characteristics based on treatment group

Variables	Surgery alone, *N* = 445	Surgery with adjuvant radiotherapy, *N* = 155	*p*-value
**Male sex**	247 (56%)	78 (50%)	0.26
**Age**	24.0 (9.0, 46.0)	40.0 (22.5, 57.5)	**< 0.001**
**Race**			0.37
White	350 (79%)	121 (78%)	
Black	66 (15%)	25 (16%)	
Asian	12 (2.7%)	7 (4.5%)	
Other	17 (3.8%)	2 (1.3%)	
**Hispanic ethnicity**	28 (6.3%)	12 (7.7%)	0.65
**Insurance status**			0.10
Private	288 (65%)	89 (57%)	
Medicaid	80 (18%)	28 (18%)	
Medicare	39 (8.8%)	26 (17%)	
Not Insured	14 (3.1%)	7 (4.5%)	
Government	8 (1.8%)	1 (0.6%)	
Missing	16 (3.6%)	4 (2.6%)	
**Income quartile**			0.28
1	82 (18%)	25 (16%)	
2	99 (22%)	31 (20%)	
3	100 (22%)	46 (30%)	
4	140 (31%)	41 (26%)	
Missing	24 (5.4%)	12 (7.7%)	
**Area of living**			0.89
Metro	310 (70%)	109 (70%)	
Urban	111 (25%)	36 (23%)	
Rural	5 (1.1%)	2 (1.3%)	
Missing	19 (4.3%)	8 (5.2%)	
**Charlson-Deyo Comorbidity index**			0.25
0	376 (84%)	123 (79%)	
1	32 (7.2%)	11 (7.1%)	
2	32 (7.2%)	19 (12%)	
3	5 (1.1%)	2 (1.3%)	
**WHO grade**			**< 0.001**
1	377 (85%)	103 (66%)	
2	68 (15%)	52 (34%)	
**Histology**			**< 0.001**
Pilocytic astrocytoma	347 (78%)	80 (52%)	
Astrocytoma, (unspecified)	69 (16%)	47 (30%)	
Fibrillary astrocytoma	14 (3.1%)	22 (14%)	
Mixed glioma	6 (1.3%)	2 (1.3%)	
Pleomorphic xanthoastrocytoma	4 (0.9%)	2 (1.3%)	
Subependymal giant cell astrocytoma	2 (0.4%)	0 (0%)	
Protoplasmic astrocytoma	1 (0.2%)	0 (0%)	
Gemistocytic astrocytom	1 (0.2%)	1 (0.6%)	
Oligodendroglioma, (unspecified)	1 (0.2%)	1 (0.6%)	
**Extent of surgical resection**			0.13
GTR	16 (3.6%)	2 (1.3%)	
STR	62 (14%)	30 (19%)	
Not specified	367 (82%)	123 (79%)	
**Adjuvant chemotherapy**	31 (7.0%)	35 (23%)	**< 0.001**
**Median days from diagnosis to adjuvant chemotherapy (IQR)**	44.0 (32.0, 80.0)	43.5 (25.0, 102.0)	0.97
**Palliative care**	3 (0.7%)	2 (1.3%)	0.61
**10-day unplanned hospital readmission**	17 (3.8%)	4 (2.6%)	0.32
**30-day postoperative mortality**	4 (0.9%)	1 (0.6%)	> 0.99
**90-day postoperative mortality**	11 (2.5%)	5 (3.2%)	0.74

**Table 2:** Additional information regarding adjuvant radiotherapy in both pre- and post-matching cohorts

Variable	Patients with adjuvant radiotherapy in the pre-matching cohort *N* = 155	Patients with adjuvant radiotherapy in the post-matching cohort *N* = 108
**Radiotherapy modality**		
Photon	97 (63%)	68 (63%)
Proton	7 (4.5%)	7 (6.5%)
Not specified	50 (32%)	33 (31%)
**Median days from diagnosis to radiotherapy (IQR)**	48.0 (32.5, 74.0)	50.0 (33.3, 79.5)
**Total radiation dose (cGy)**	5,040 (4,500, 5,040)	5,040 (4,500, 5,040)

There was no significant difference in sex distribution between the two groups (*p* = 0.26), with 247 patients (56%) being male in the surgery alone group compared to 78 patients (50%) in the surgery with adjuvant radiotherapy group. However, patients in the surgery with adjuvant radiotherapy group were significantly older, with a median age of 40.0 years (IQR: 22.5, 57.5) compared to 24.0 years (IQR: 9.0, 46.0) in the surgery alone group (*p* < 0.001).

Racial and ethnic distributions did not differ significantly between the groups (*p* = 0.37 and *p* = 0.65, respectively), with 79% vs. 78% White, 15% vs. 16% Black, 2.7% vs. 4.5% Asian, and 6.3% vs. 7.7% Hispanic among the surgery alone vs. surgery with adjuvant radiotherapy groups. Insurance status did not show any significant difference between the groups (*p* = 0.10), with most patients having private insurance (65% vs. 57%), followed by Medicaid (18% vs. 18%), and Medicare (8.8% vs. 17%). No differences were found in terms of income quartile distribution (*p* = 0.28).

In both groups, most patients lived in metropolitan areas (70% vs. 70%), followed by urban (25% vs. 23%), and rural areas (1.1% vs. 1.3), with no significant difference between the groups (*p* = 0.89). The Charlson-Deyo comorbidity index was similar between the groups (*p* = 0.25), with most of the patients having a comorbidity index of 0 in the surgery alone group and surgery with adjuvant radiotherapy group (84% vs. 79%).

WHO grade was significantly different between the groups (*p* < 0.001). In the surgery alone group, 377 patients (85%) had a WHO grade of 1, and 68 patients (15%) had a WHO grade of 2. In the surgery with adjuvant radiotherapy group, 103 patients (66%) had a WHO grade of 1, and 52 patients (34%) had a WHO grade of 2.

Similarly, histological subtypes were significantly different between the groups (*p* < 0.001). In the surgery alone group, the two most prevalent subtypes were pilocytic astrocytoma with 347 patients (78%) and low-grade astrocytomas of unspecified histology with 69 patients (16%). In the surgery with adjuvant radiotherapy group, the two most prevalent subtypes were also pilocytic astrocytoma with 80 patients (52%) and low-grade astrocytomas of unspecified histology with 47 patients (30%).

There were no significant differences in the extent of surgical resection between the groups (*p* = 0.13). In the surgery alone group, 16 patients (3.6%) had a gross total resection (GTR), 62 (14%) had a subtotal resection (STR), and 367 (82%) had unspecified extent of resection. In the surgery with adjuvant radiotherapy group, 2 patients (1.3%) had a GTR, 30 (19%) had an STR, and 123 (79%) had unspecified resection.

Adjuvant chemotherapy was administered to 31 patients (7.0%) in the surgery alone group and 35 patients (23%) in the surgery with adjuvant radiotherapy group (*p* < 0.001). The median number of days from diagnosis to adjuvant chemotherapy was 44.0 days (IQR: 32.0, 80.0) in the surgery alone group and 43.5 days (IQR: 25.0, 102.0) in the surgery with adjuvant radiotherapy group (*p* = 0.97).

Palliative care was provided to 3 patients (0.7%) in the surgery alone group and 2 patients (1.3%) in the surgery with adjuvant radiotherapy group (*p* = 0.61). The rate of 10-day unplanned hospital readmission was similar in both surgery alone and surgery with adjuvant radiotherapy groups (3.8% vs. 2.6%; *p* = 0.32). The 30- and 90-day postoperative mortality rate were 0.9% and 2.5% in the surgery alone group and 0.6% and 3.2% in the surgery with adjuvant radiotherapy group, with no significant differences (*p* > 0.99 and *p* = 0.74, respectively).

### Radiotherapy modality

In the pre-matching cohort, 97 patients (63%) received photon radiotherapy, 50 patients (32%) had an unspecified radiotherapy modality, and 7 patients (4.5%) received proton radiotherapy. The median number of days from diagnosis to radiotherapy was 48.0 days (IQR: 32.5, 74.0). The total radiation dose was 5,040 cGy (IQR: 4,500, 5,040).

In the post-matching cohort, 68 patients (63%) received photon radiotherapy, 33 patients (31%) had an unspecified radiotherapy modality, and 7 patients (6.5%) received proton radiotherapy. The median number of days from diagnosis to radiotherapy was 50.0 days (IQR: 33.3, 79.5). The total radiation dose was 5,040 cGy (IQR: 4,500, 5,040).

### Survival outcomes

**Fig. 2:**
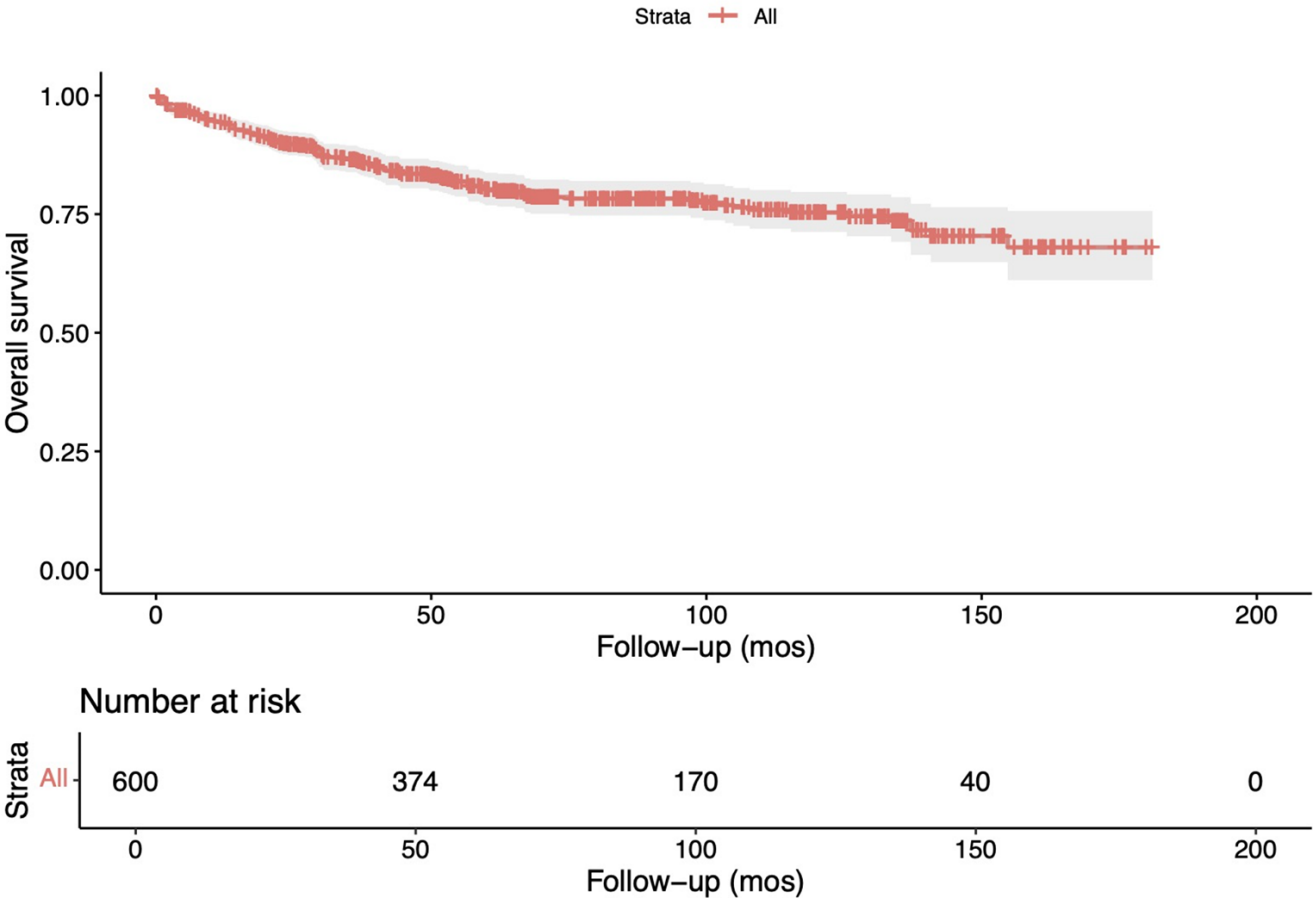
Overall survival among the entire cohort of patients with spinal low-grade gliomas undergoing either surgery alone or surgery with adjuvant radiotherapy

**Fig. 3:**
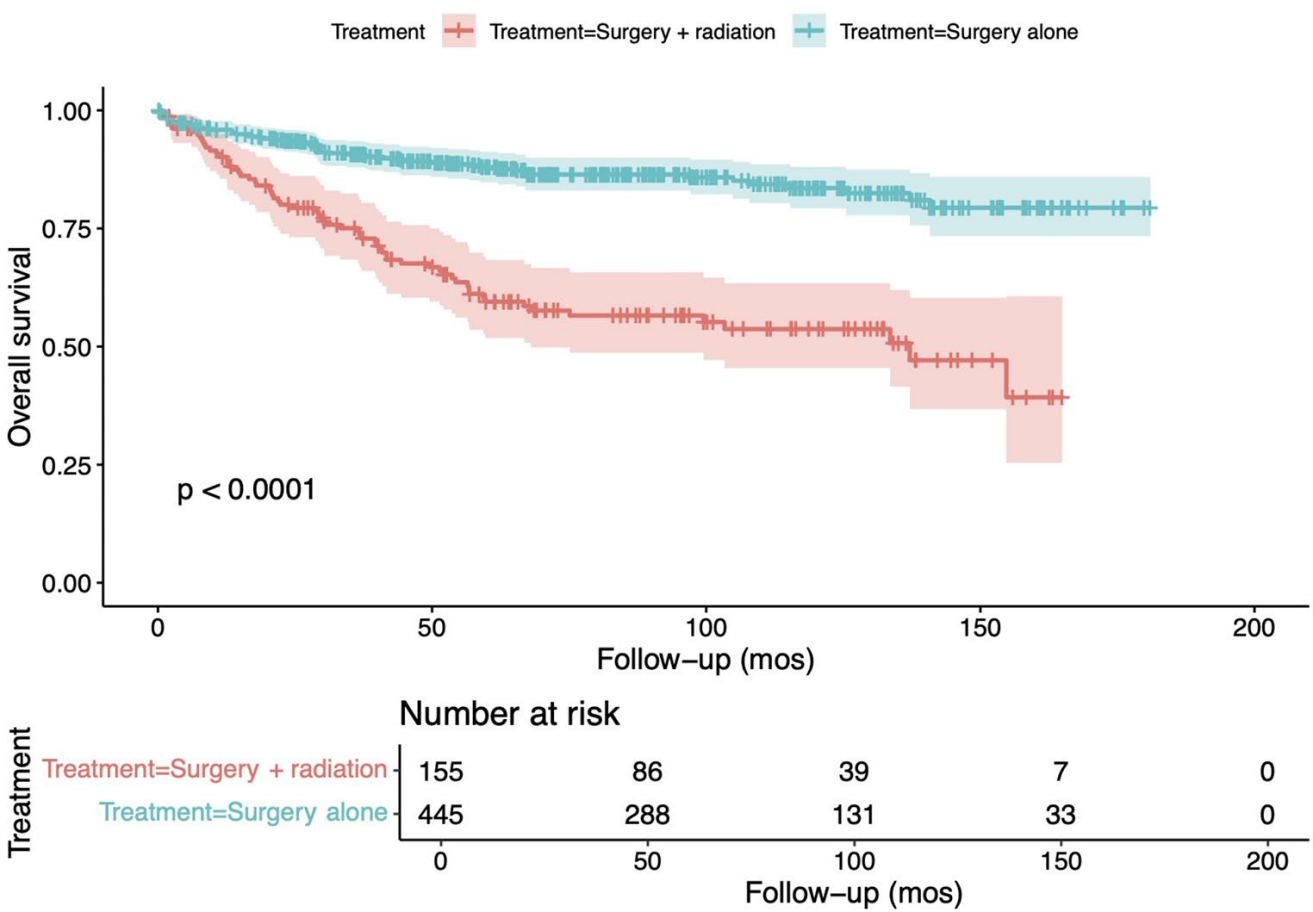
Overall survival among patients with spinal low-grade gliomas undergoing surgery alone versus surgery with adjuvant radiotherapy, prior to propensity score matching

On survival analysis, the observed overall 5-year survival rate for the entire cohort reached around 80%, short of 75% at 10-years (Fig. 2). Survival analysis following stratificaiton of the cohort into surgery alone and surgery with adjuvant radiotherapy groups revealed a significantly higher mortality in patients who received adjuvant radiotherapy (*p* < 0.0001; Fig. 3).

**Fig. 4:**
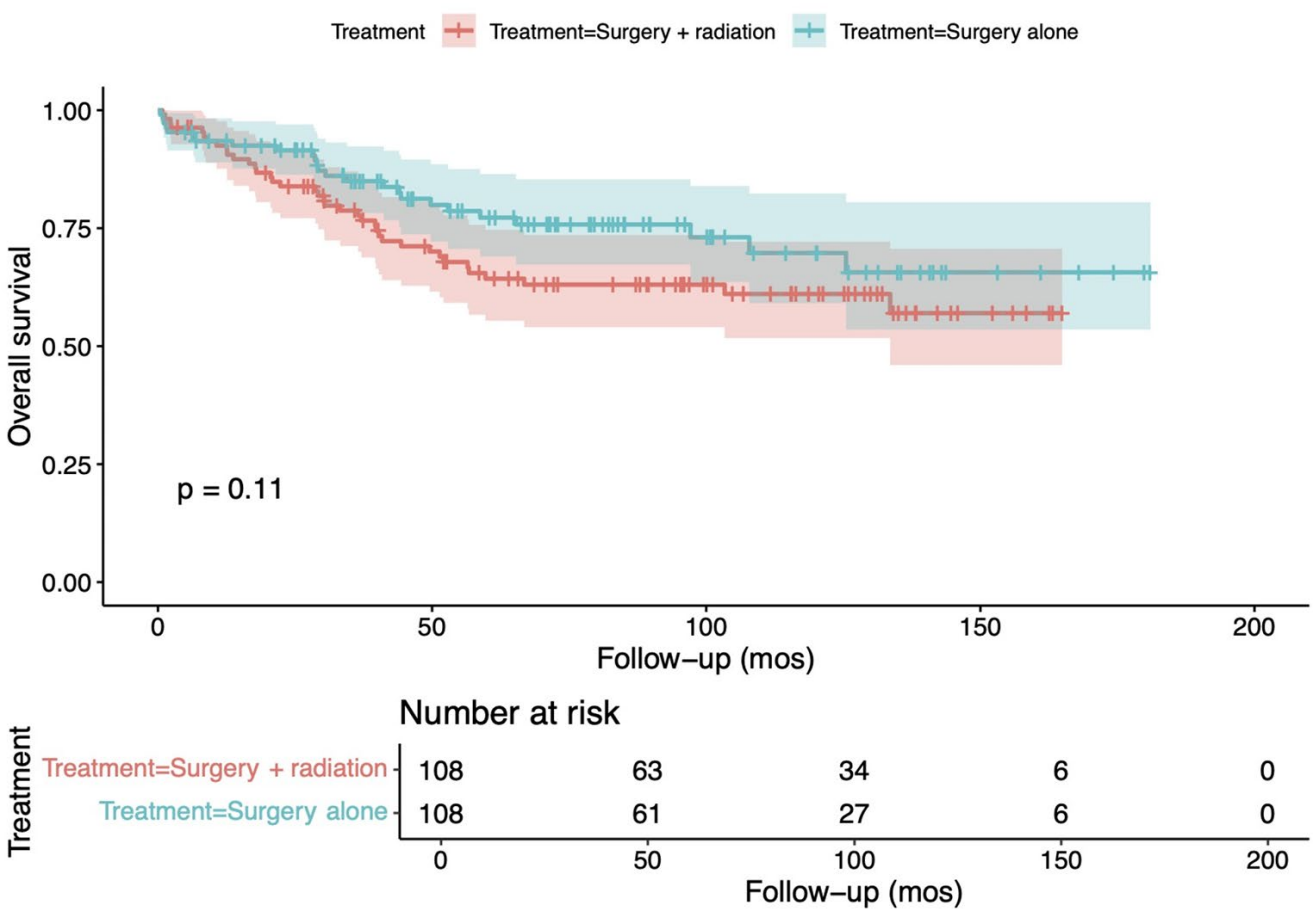
Overall survival among patients with spinal low-grade gliomas undergoing surgery alone versus surgery with adjuvant radiotherapy, following propensity score matching

Following propensity score matching to account for potential confounders (Supplementary Figure A and Table A), the difference in overall survival among the groups was no longer significant (*p* = 0.11; Fig. 4).

## Discussion

Adjuvant radiotherapy is reportedly part of the standard of care in brain low grade gliomas, with existing studies showing 50.4–54 Gy as the most common radiation regimen [9]. On the other hand, the use of adjuvant radiotherapy in the management of sLGG is more controversial, and the response of this specific subgroup of gliomas to radiotherapy has been poorly studied. This study reports on the overall survival outcomes associated with the use of adjuvant radiotherapy for sLGG in a nationwide US-based cohort. The results of this study seem to indicate a lack of added benefit in terms of overall survival associated with the use of adjuvant radiotherapy for sLGG both prior to and after propensity score matching.

In the initial cohort, prior to propensity score matching, as a result of the Berkson bias, patients receiving adjuvant radiotherapy were found to have more advanced disease, as pointed by the overrepresentation of patients with WHO grade 2, as opposed to grade 1 tumors as well as those receiving concomitant chemotherapy in that cohort, as compared to the cohort of patients receiving surgery alone. This is also reflected by the significantly higher overall mortality among patients receiving both surgery and adjuvant radiotherapy when compared to those only receiving surgery (*p* < 0.0001; Fig. 3) [10].

After adjusting for confounding variables using propensity score matching, the difference was no longer significant, suggesting that overall survival outcomes are similar regardless of whether adjuvant radiotherapy is administered.

Previous studies in the limitedly available literature corroborate these findings. For instance, one study using a different US-based nationwide database, the SEER database, demonstrated no significant difference in overall survival outcomes with the administration of adjuvant radiotherapy for sLGG [11]. In addition, another study from Japan on WHO Grade II spinal astrocytomas, reported a lack of a significant difference in both overall and progression-free survival outcomes among patients receiving surgery alone when compared to those receiving surgery with adjuvant radiotherapy [12]. In addition, the authors found performance scores in both groups to gradually decrease after operation. Interestingly, the deterioration was more notable among patients receiving adjuvant radiotherapy [12].

On the other hand, in a study by Abdel-Wahab et al., the authors found a significant progression-free survival benefit associated with the use of adjuvant radiotherapy in patients with spinal gliomas. However, their study included spinal gliomas of WHO grades 2, 3, and 4, which may indicate a potential benefit of adjuvant radiotherapy seen in higher grade spinal gliomas.

Along these lines, another study showed that adjuvant radiation improved survival compared with no radiation in both spinal glioblastoma (*p* = 0.048) and WHO grade 3 tumors (*p* = 0.023) [13]. However, in a study by Fakhreddine et al. on infiltrative spinal gliomas, including tumors of WHO grades 2, 3, and 4, adjuvant radiotherapy did not significantly influence survival outcomes [14].

The evidence surrounding the safety and usefulness of adjuvant radiotherapy in the context of spinal gliomas remains uncertain. Radiation therapy targeting the spinal cord carries significant risks, including the potential for further damage to already compromised neural tissue, which may result in detrimental and potentially irreversible complications [15–17]. The risk of these adverse effects ought to be carefully weighed against the therapeutic benefits [18], particularly in cases where the spinal cord is already compromised by both the tumor and the subsequent surgical treatment. In the context of spinal gliomas, this concept is evidenced by the accentuated performance deterioration seen in patients receiving surgery with radiation therapy as compared to those only receiving surgery, in the Japanese study by Kanematsu et al. [12].

While the aggressive nature of high-grade spinal gliomas (WHO grades 3 and 4) may justify the risks and the threat to patient quality-of-life associated with adjuvant radiotherapy, this rationale does not seem to equally apply in the context of sLGG (WHO grades 1 and 2). As such, based on the current study, adjuvant radiotherapy was not associated with any survival benefits in the treatment of sLGG. Larger multicentric studies with higher levels of evidence are required to validate these findings and avoid the potential risks of exposing patients to unnecessary procedures.

### Limitations

This study has several limitations, mainly inherent to database studies in general [19, 20]. First, several patients with missing data had to be excluded, which may have biased the results. The absence of data pertaining to the extent of tumor resection, histological subtypes, and other relevant variables limited the conduction of more comprehensive matching and subgroup-analysis. While matching accounted for all potential cofounders that were available in the database, some other unmeasured confounders may still have an impact on the outcomes of this study, which raises certain concerns with respect to its internal validity. This underscores the need for more rigorous validating studies. The NCDB has several limitations, including data missingness and the absence of baseline patient information, such as detailed comorbidity data or other information on the exact indications of the use of adjuvant therapy. In the current study, missingness of data was especially marked in the context of extent of tumor resection, which greatly limited our ability to account for this factor in the analysis. Additionally, the NCDB does not include neither tumor recurrence data nor patient-reported outcomes or health-related quality-of-life measures. As such, this study only investigated overall survival as a primary outcome. Consequently, although highly unlikely as highlighted in previous studies, the current study cannot preclude a progression-free survival benefit of adjuvant radiotherapy.

## Conclusion

In conclusion, this study found no significant overall survival benefit associated with the use of adjuvant radiotherapy for spinal low-grade gliomas (sLGG). Although patients who received adjuvant radiotherapy initially demonstrated higher mortality rates, this difference was largely due to confounding factors such as more advanced disease in this group. After adjusting for these variables through propensity score matching, survival outcomes between groups became comparable, suggesting that adjuvant radiotherapy may not offer a clear survival advantage for patients with sLGG. While these findings align with previous studies, larger, multicentric studies are still needed to better define the role of adjuvant radiotherapy in sLGG management and to ensure that patients are not exposed to unnecessary risks.

## Electronic supplementary material

Below is the link to the electronic supplementary material.

Supplementary Material 1

Supplementary Material 2

## Data Availability

No datasets were generated or analysed during the current study.
