# Supplementary figures and images for: The impact of adjuvant radiotherapy on overall survival in spinal low-grade gliomas: a propensity score-matched analysis
Source: J Neurooncol. 2024 Nov 11;171(3):629–36. doi: 10.1007/s11060-024-04880-3 (PMC11729206; doi:10.1007/s11060-024-04880-3)

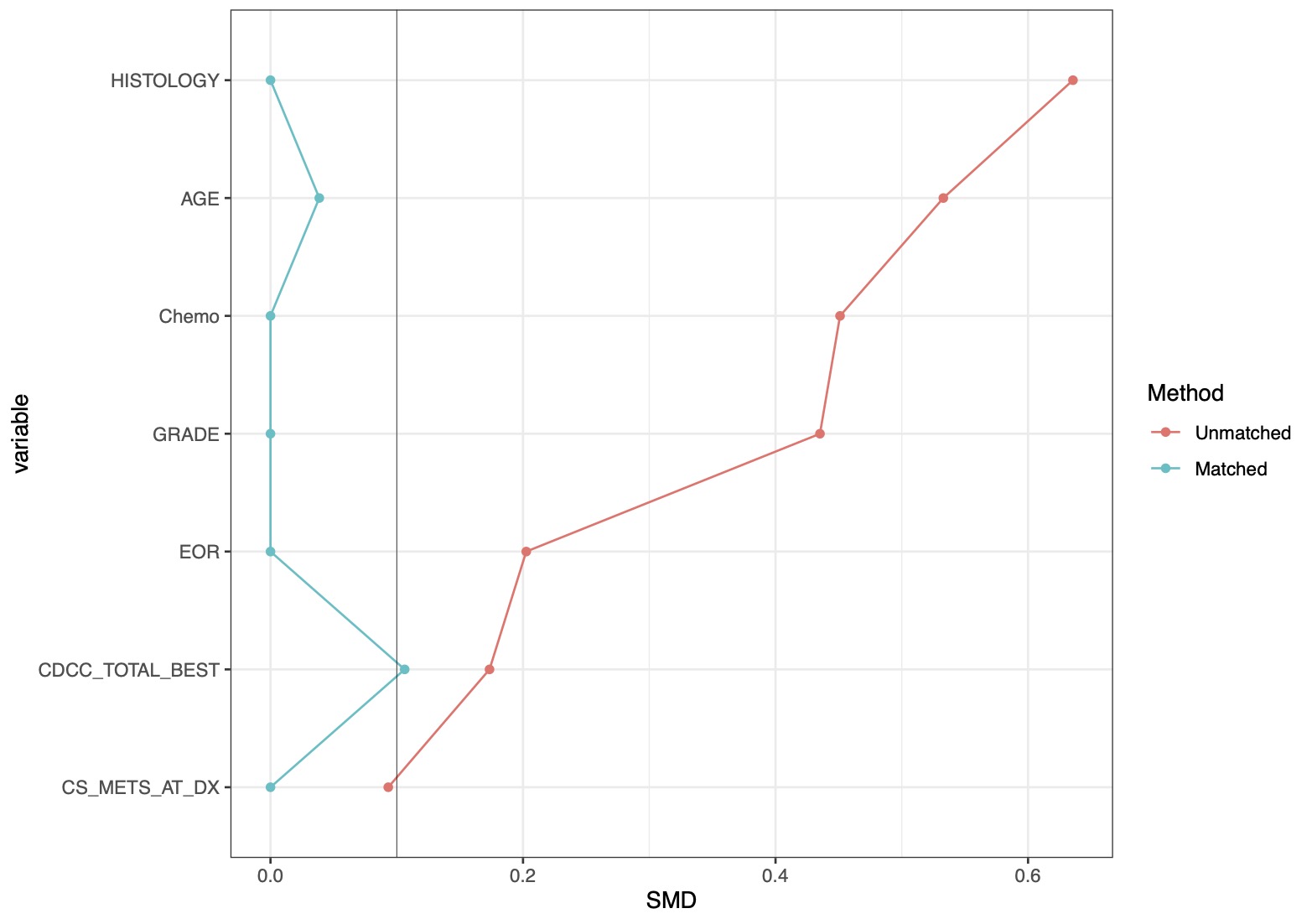

Supplement: Supplementary file 1 — Supplementary Material 1 [file 11060_2024_4880_MOESM1_ESM.jpg]
