# Supplementary material for: The impact of adjuvant radiotherapy on overall survival in spinal low-grade gliomas: a propensity score-matched analysis
Source: J Neurooncol. 2024 Nov 11;171(3):629–36. doi: 10.1007/s11060-024-04880-3 (PMC11729206; doi:10.1007/s11060-024-04880-3)
Supplement: Supplementary file 2 — Supplementary Material 2 [file 11060_2024_4880_MOESM2_ESM.docx]

| Variables | **Surgery alone**,  N = 108 | **Surgery with adjuvant radiotherapy**,  N = 108 | **p-value** |
| --- | --- | --- | --- |
| **Male sex** | 56 (52%) | 54 (50%) | 0.79 |
| **Age (IQR)** | 37.0 (16.3, 53.3) | 32.0 (18.8, 54.0) | 0.68 |
| **Race** |  |  | 0.61 |
| White | 89 (82%) | 85 (79%) |  |
| Black | 14 (13%) | 18 (17%) |  |
| Asian | 2 (1.9%) | 3 (2.8%) |  |
| Other | 3 (2.8%) | 2 (1.9%) |  |
| **Hispanic ethnicity** | 9 (8.3%) | 7 (6.5%) | 0.58 |
| **Insurance status** |  |  | 0.92 |
| Private | 67 (62%) | 62 (57%) |  |
| Medicaid | 16 (15%) | 20 (19%) |  |
| Medicare | 15 (14%) | 17 (16%) |  |
| Not Insured | 8 (7.4%) | 6 (5.6%) |  |
| Government | 1 (0.9%) | 1 (0.9%) |  |
| Missing | 1 (0.9%) | 2 (1.9%) |  |
| **Income quartile** |  |  | 0.56 |
| 1 | 24 (22%) | 16 (15%) |  |
| 2 | 21 (19%) | 24 (22%) |  |
| 3 | 27 (25%) | 35 (32%) |  |
| 4 | 30 (28%) | 27 (25%) |  |
| Missing | 6 (5.6%) | 6 (5.6%) |  |
| **Area of living** |  |  | 0.58 |
| Metro | 74 (69%) | 79 (73%) |  |
| Urban | 27 (25%) | 23 (21%) |  |
| Rural | 2 (1.9%) | 0 (0%) |  |
| Missing | 5 (4.6%) | 6 (5.6%) |  |
| **Charlson-Deyo Comorbidity index** |  |  | 0.92 |
| 0 | 91 (84%) | 89 (82%) |  |
| 1 | 5 (4.6%) | 7 (6.5%) |  |
| 2 | 9 (8.3%) | 10 (9.3%) |  |
| 3 | 3 (2.8%) | 2 (1.9%) |  |
| **WHO grade** |  |  | >0.99 |
| 1 | 79 (73%) | 79 (73%) |  |
| 2 | 29 (27%) | 29 (27%) |  |
| **Histology** |  |  | >0.99 |
| Protoplasmic astrocytoma | 0 (0%) | 0 (0%) |  |
| Astrocytoma, NOS | 35 (32%) | 35 (32%) |  |
| Fibrillary astrocytoma | 6 (5.6%) | 6 (5.6%) |  |
| Gemistocytic astrocytom | 1 (0.9%) | 1 (0.9%) |  |
| Mixed glioma | 1 (0.9%) | 1 (0.9%) |  |
| Oligodendroglioma, NOS | 0 (0%) | 0 (0%) |  |
| Pilocytic astrocytoma | 65 (60%) | 65 (60%) |  |
| Pleomorphic xanthoastrocytoma | 0 (0%) | 0 (0%) |  |
| Subependymal giant cell astrocytoma | 0 (0%) | 0 (0%) |  |
| **Extent of surgical resection** |  |  | >0.99 |
| GTR | 2 (1.9%) | 2 (1.9%) |  |
| STR | 16 (15%) | 16 (15%) |  |
| Not specified | 90 (83%) | 90 (83%) |  |
| **Median days from diagnosis to surgery (IQR)** | 0.5 (0.0, 14.5) | 0.0 (0.0, 5.0) | **0.031** |
| **Adjuvant chemotherapy** | 6 (5.6%) | 6 (5.6%) | >0.99 |
| **Median days from diagnosis to adjuvant chemotherapy (IQR)** | 69.0 (15.5, 150.3) | 25.0 (11.0, 109.0) | 0.79 |
| **Palliative care** | 1 (0.9%) | 2 (1.9%) | >0.99 |
| **10-day unplanned hospital readmission** | 3 (2.8%) | 2 (1.9%) | >0.99 |
| **30-day postoperative mortality** | 3 (2.8%) | 1 (0.9%) | 0.76 |
| **90-day postoperative mortality** | 5 (4.6%) | 3 (2.8%) | 0.82 |
